# Supplementary figures and images for: Robustness analysis of metabolic predictions in algal microbial communities based on different annotation pipelines
Source: PeerJ. 2021 May 6;9:e11344. doi: 10.7717/peerj.11344 (PMC8106915; doi:10.7717/peerj.11344)

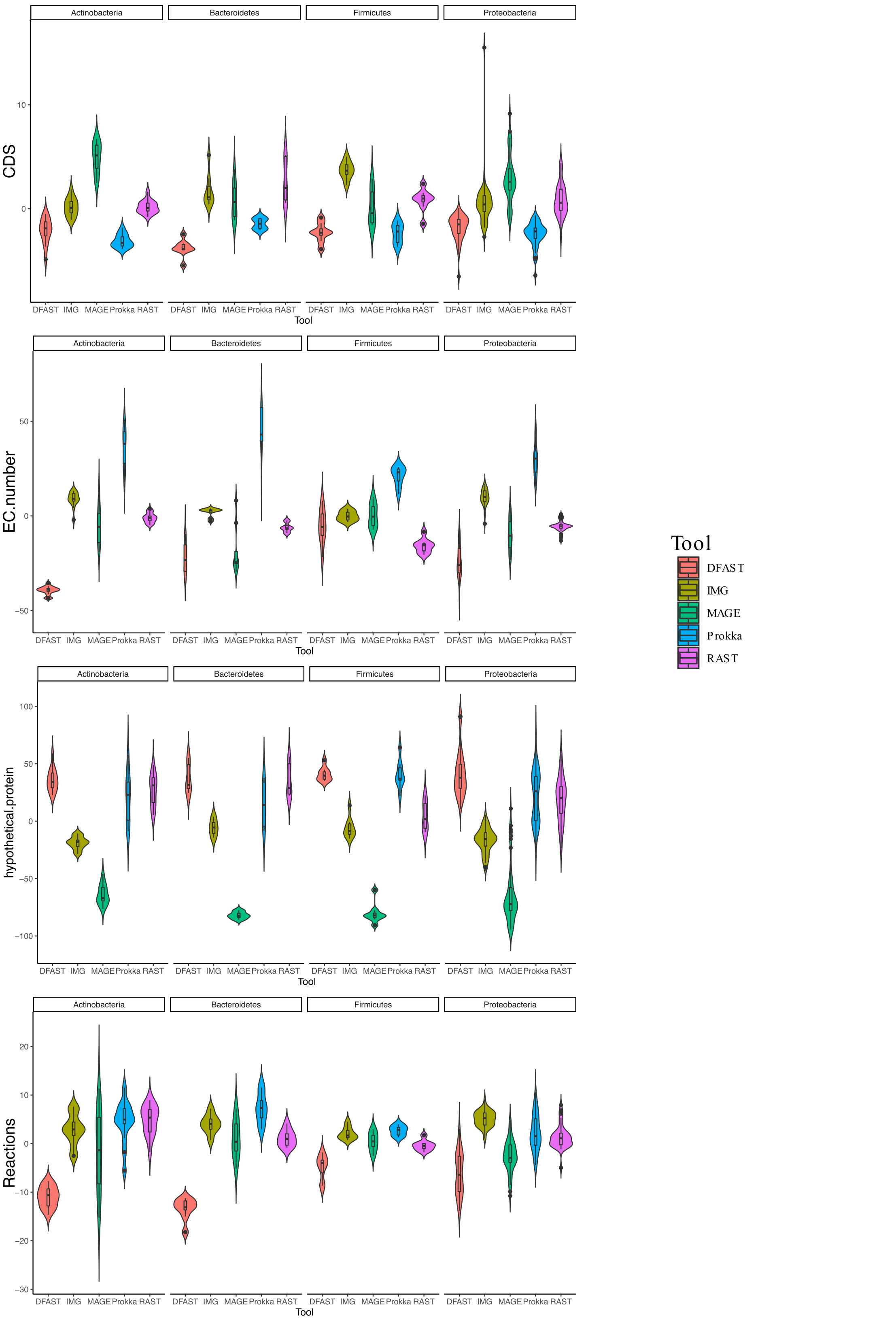

Supplement: Supplemental Information 2 — The graphs show the deviation of each genome annotated with everyone pipeline from the mean across all pipelines in %. [file peerj-09-11344-s002.png]
